# Acupuncture improves blood–brain barrier integrity through multi-targeted mechanisms: a preclinical meta-analysis

**DOI:** 10.3389/fneur.2025.1648117

**Published:** 2025-11-07

**Authors:** Kexing Zhang, Ying Liang, Yinxin Wang, Tong Yin, Kangwen Ming

**Affiliations:** 1 Medical College of Acupuncture-Moxibustion and Rehabilitation, Guangzhou University of Chinese Medicine, Guangzhou, China; 2 Guangzhou University of Chinese Medicine, Guangzhou, China; 3 Department of Acupuncture and Rehabilitation, The Affiliated Traditional Chinese Medicine Hospital of Guangzhou Medical University, Guangzhou, China

**Keywords:** acupuncture, blood–brain barrier, tight junction, neuroinflammation, meta-analysis

## Abstract

**Objective:**

The objective of this study is to assess the impact of acupuncture regarding Blood–Brain barrier (BBB) permeability and provide a data foundation for clinical practice.

**Methods:**

A database search was carried out in PubMed, Embase, Cochrane Library, and Web of Science to collect controlled animal experiments that investigated the impact of acupuncture on BBB permeability. BBB permeability was primarily assessed by indicators such as Evans Blue (EB) extravasation. The SYRCLE risk-of-bias tool was utilized to assess the quality of the comprised studies, and statistical software was employed for data evaluation. For continuous outcomes, a random-effects model was used to calculate pooled standardized mean differences (SMD) with 95% confidence intervals (CI), and heterogeneity was quantified using the *I*^2^ statistic.

**Results:**

Thirty-two papers were incorporated. Outcomes from the meta-analysis showed that acupuncture significantly reduced the EB leakage in brain tissue (SMD = −0.65, 95% CI [−0.94, −0.37], *p* < 0.001), indicating its effectiveness in improving BBB permeability. Additionally, acupuncture upregulated the levels of occludin, claudin-5, and ZO-1, inhibited the activity of matrix metalloproteinase-9 (MMP-9), reduced the amounts of glial activation markers (Iba-1, GFAP) and inflammatory mediators (IL-1β, IL-6, TNF-α), and regulated the levels of aquaporin-4 (AQP4).

**Conclusion:**

acupuncture may improve BBB integrity by means of multiple mechanisms, including the enhancement of tight junction protein production in endothelial cells, inhibiting MMP-9 mediated extracellular matrix (ECM) degradation, modulating glial cell activation and inflammatory responses, and downregulating AQP4-dependent edema.

**Systematic review registration:**

https://inplasy.com/inplasy-2025-2-0102/, identifier: INPLASY202520102.

## 1 Introduction

The Blood–Brain barrier (BBB), a highly selective barrier that exists between the central nervous system (CNS) and the peripheral circulation. It is mainly composed of brain microvascular endothelial cells, tight junctions, the basement membrane, pericytes, and astrocytes. The main role of the BBB is to preserve homeostasis of the CNS, protect brain tissue from toxic substances, and ensure the supply of essential molecules required for neuronal activity (1). The BBB primarily relies on tight junctions (TJs) between endothelial cells. These junction complexes consist of transmembrane proteins (claudin-5, occludin, tricellulin, marvelD3) and cytoplasmic membrane-associated proteins (ZO-1, ZO-2, ZO-3) (2). Claudin-5, as a BBB-specific transmembrane protein, maintains intercellular barrier integrity by forming tightly sealed transmembrane strands (3). Matrix metalloproteinases (MMPs), particularly MMP-9, regulate BBB permeability by degrading extracellular matrix and tight junction proteins. Abnormal elevation of their activity constitutes a key mechanism in BBB damage (4). Overexpression of glial cell activation markers Iba-1 (microglia) and GFAP (astrocytes) drives neuroinflammatory responses. These markers disrupt endothelial tight junctions through the release of pro-inflammatory factors (IL-1β, IL-6, TNF-α), ultimately increasing BBB permeability (5, 6). Aquaporin 4 (AQP4) is primarily distributed in astrocyte foot processes, and its abnormal expression is closely associated with cerebral edema formation and BBB dysfunction (7). Notably, breakdown of the BBB is frequently seen in a range of neurological disorders, such as stroke, Alzheimer's disease, and Parkinson's disease, and is strongly linked to the onset and progression of these diseases (8).

Acupuncture, a classic Chinese therapeutic technique, has demonstrated distinct advantages in clinical interventions for neurological diseases due to its simplicity, safety, and low risk of side effects (9, 10). As a classic treatment technology of traditional Chinese medicine, acupuncture shows unique advantages in the clinical intervention of nervous system diseases, and its mechanism involves the coordinated regulation of multiple molecular targets. Recent studies have demonstrated that acupuncture can influence BBB permeability through multiple mechanisms. Specifically, acupuncture reduces LPS, TNF-α, and IL-1β levels while positively regulating intestinal flora and addressing Blood–Brain barrier dysfunction (11). Additionally, acupuncture inhibits Blood–Brain barrier damage by modulating autophagy-apoptosis balance (12). Furthermore, acupuncture suppresses the ERK1/2-Cx43 signaling cascade, thereby alleviating astrocyte-mediated neurotoxicity. Promoting Blood–Brain Barrier Recovery (13). Recent research indicates that acupuncture may influence the permeability of BBB through multiple pathways, for example, bloodletting at the twelve well points of the hand improves BBB permeability by downregulating occludin and claudin-5 (14), while Acupuncture modulates the levels of metalloproteinase isoforms (MMP-2), transmembrane water channel proteins (AQP4, AQP9), and inflammatory cell infiltration in a rat model of middle cerebral artery occlusion (MCAO) (15).

Although a number of researches have explored the impact of acupuncture concerning BBB permeability, the findings derived from these experiments exhibit certain discrepancies, which present challenges for the clinical translation of acupuncture's impact on BBB permeability. First, inconsistencies in research findings: Significant variations exist among studies regarding acupuncture parameters (acupoint selection, stimulation frequency, treatment duration), animal models (ischemic stroke, traumatic brain injury, neurodegenerative diseases), and biomarkers, leading to inconclusive conclusions. Second, lack of systematic mechanism analysis: Current research predominantly focuses on single-target or pathway investigations, lacking comprehensive understanding of acupuncture's multi-target synergistic mechanisms. Third, insufficient clinical translation evidence: A substantial gap persists between preclinical studies and clinical practice, with unclear optimal parameters and indications for acupuncture's protective effects on Blood–Brain barrier (BBB) permeability. To date, no systematic meta-analysis has been conducted to improve BBB permeability through acupuncture, leaving the evidence base inadequate for evidence-based medical practice (16). Thus, this study strives to integrate existing preclinical experiments and evaluate acupuncture's effects on BBB permeability through meta-analysis, while also delving into the potential mechanisms, with the goal of offering a theoretical basis and scientific support for acupuncture's application in the therapy of neurological diseases.

## 2 Methods

This study follows the PRISMA guidelines (17) for Animal Research Systematic Reviews and Meta-Analyses, and was prospectively registered on the INPLASY platform on February 22, 2025 (Registration ID: INPLASY202520102) and can be accessed via the link https://doi.org/10.37766/inplasy2025.2.0102.

### 2.1 Database search strategy

The following electronic reference databases were searched: PubMed, Embase, Cochrane Library, and Web of Science, with the database updated until December 2024. The main search terms are as follows: [(Acupuncture) OR (Acupuncture Therapy) OR (Acupuncture, Ear) OR (Acupuncture Points)] AND (Blood–Brain Barrier), as shown in Table 1 (For other database retrieval strategies, please refer to Supplementary Tables 1–3).

**Table 1:** Search strategy on PubMed.

**#1**	**(((Acupuncture[MeSH Terms]) OR (Acupuncture Therapy[MeSH Terms])) OR (Acupuncture, Ear[MeSH Terms])) OR (Acupuncture Points[MeSH Terms])**
#2	((((((((((((((((((((((((((((((Acupuncture) OR (Acupuncture Therapy)) OR (Pharmacopuncture)) OR (Acupuncture Treatment)) OR (Acupuncture Treatments)) OR (Treatment, Acupuncture)) OR (Therapy, Acupuncture)) OR (Pharmacoacupuncture Treatment)) OR (Treatment, Pharmacoacupuncture)) OR (Pharmacoacupuncture Therapy)) OR (Therapy, Pharmacoacupuncture)) OR (Acupotomy)) OR (Acupotomies)) OR (Acupuncture, Ear)) OR (Acupunctures, Ear)) OR (Ear Acupunctures)) OR (Acupuncture, Auricular)) OR (Acupunctures, Auricular)) OR (Auricular Acupunctures)) OR (Auricular Acupuncture)) OR (Ear Acupuncture)) OR (Acupuncture Points)) OR (Acupuncture Point)) OR (Point, Acupuncture)) OR (Points, Acupuncture)) OR (Acupoints)) OR (Acupoint)) OR (Acupuncture Analgesia)) OR (Analgesia, Acupuncture)) OR (Acupuncture Anesthesia)) OR (Anesthesia, Acupuncture)
#3	(#1) OR (#2)
#4	Blood–Brain Barrier[MeSH Terms]
#5	((((((((((((((Blood–Brain Barrier) OR (Barrier, Blood–Brain)) OR (Barriers, Blood–Brain)) OR (Blood Brain Barrier)) OR (Blood–Brain Barriers)) OR (Hemato-Encephalic Barrier)) OR (Barrier, Hemato-Encephalic)) OR (Barriers, Hemato-Encephalic)) OR (Hemato Encephalic Barrier)) OR (Hemato-Encephalic Barriers)) OR (Brain-Blood Barrier)) OR (Barrier, Brain-Blood)) OR (Barriers, Brain-Blood)) OR (Brain Blood Barrier)) OR (Brain-Blood Barriers)
#6	(#4) OR (#5)
#7	(#3) AND (#6)

### 2.2 Inclusion criteria

(1) Controlled experiment; (2) Rodent models (mice and rats) were considered (18), with no restrictions on sex, age, or weight; (3) The experimental group received acupuncture intervention and/or acupuncture pre-intervention, while the control group received carrier solutions, isotonic saline, sham acupuncture, clinically proven effective drugs, or no intervention;(4) Studies must include outcome measures related to Blood–Brain barrier permeability, such as indicators of BBB integrity, tight junction proteins, matrix metalloproteinases, glial activation markers, inflammatory mediators, or water channel proteins.

### 2.3 Exclusion criteria

(1) Uncontrolled studies; (2)Studies that did not use rodent models (mice and rats); (3) Reviews, meta-analyses, conference abstracts, editorials, and letters; (4) Research not involving acupuncture intervention or acupuncture pre-intervention; (5) Studies for which data could not be obtained; (6) Studies that did not include any outcome measures related to Blood–Brain barrier permeability were excluded.

### 2.4 Study screening

EndNote was used for literature screening and exclusion. Initially, 2 investigators independently conducted a preliminary screening of the titles to exclude duplicate studies, non-controlled trials, reviews, conference papers, protocols, editorials, and communications. Next, the two researchers performed an abstract review, assessing the abstracts to determine eligible studies for inclusion and reject those that do not satisfy the inclusion criteria. Finally, the 2 investigators conducted a full-text review, reading the studies left in their entirety and further determining their inclusion. During this phase, 2 investigators autonomously assessed the publications, the leftover studies were compared. If consensus was reached, the study was included. In case of disagreement, a third researcher was consulted to discuss and resolve the discrepancy.

### 2.5 Data extraction

Two investigators conducted data extraction independently. A predefined data collection template was employed to document the data included in the study, which consisted of the researcher's name, year of release, nation of experimentation, experimental animal strain, gender, weight, age, disease model type, acupuncture points, acupuncture parameters, duration of each intervention, total number of interventions, and primary outcomes.

### 2.6 Quality assessment

The Laboratory Animal Experimentation (SYRCLE) tool was used by two investigators to independently assess the risk of bias in the included studies. This instrument includes ten domains: (1) Randomized sequence generation, (2) Similarity in baseline characteristics, (3) Concealing group assignment, (4) Randomization of animal housing, (5) Blinding of researchers, (6) Result evaluation with randomization, (7) Blinded result assessment, (8) Missing data reporting, (9) Result reporting bias, and (10) Other biases. Risk of bias in the assessment was classified as “Low,” “High,” or “Unclear”.

### 2.7 Data analysis

Data synthesis for the meta-analysis, including generating forest plots, was conducted using Review Manager (RevMan, version 5.4). Additional statistical assessments, such as sensitivity analysis and funnel plot creation, were performed using STATA (version 15.1). The standardized mean difference (SMD) and 95% confidence ranges (95% CI) were used to describe continuous variables, and when measurement units differed across studies, the standardized mean difference (SMD) was employed for standardization. Heterogeneity between Studies are evaluated with *I*^2^ and Cochran's Q test. Sensitivity analysis is performed using the single-study exclusion approach to test the robustness of the analysis. Publication bias analysis was conducted if the study count reached 10 or more. Funnel plot examination, egger's regression analysis, begg's correlation test and sensitivity analysis were used to assess potential publication bias in the systematic review.

## 3 Results

### 3.1 Study eligibility screening process

**Figure 1:**
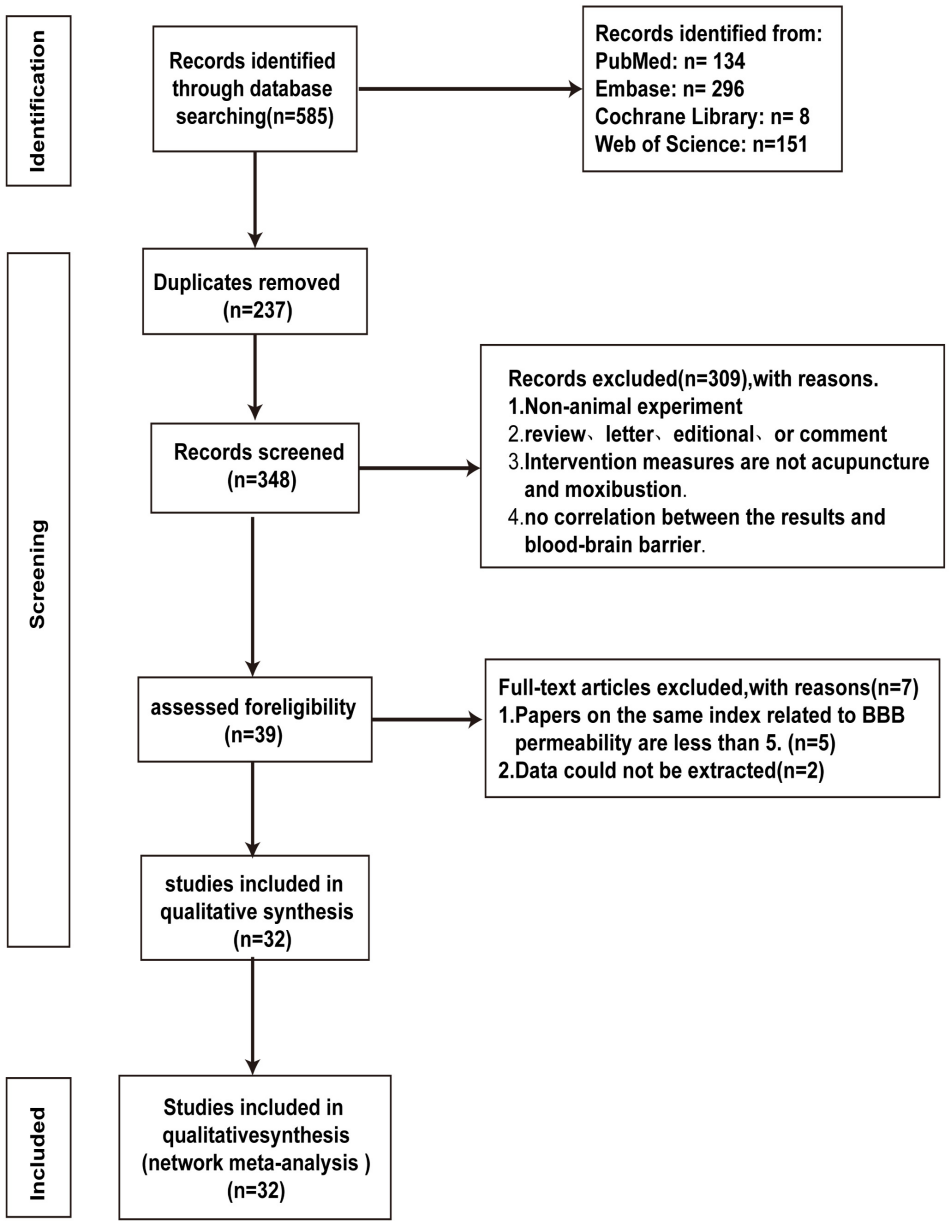
PRISMA flow diagram.

Five hundred and eighty five articles were collected from electronic databases. Once duplicates were eliminated, we screened the titles and abstracts of the 348 remaining articles, which led to the exclusion of 309 articles. We then read the full texts of the remaining 39 articles, and 7 articles were excluded (reasons for exclusion: data could not be extracted or the number of studies with the same outcome measures was less than five). Finally, 32 articles were included incorporated into this study (11, 12, 14, 15, 19–46) (Figure 1).

### 3.2 Study characteristics and outcome determination

#### 3.2.1 Study characteristics

**Figure 2:**
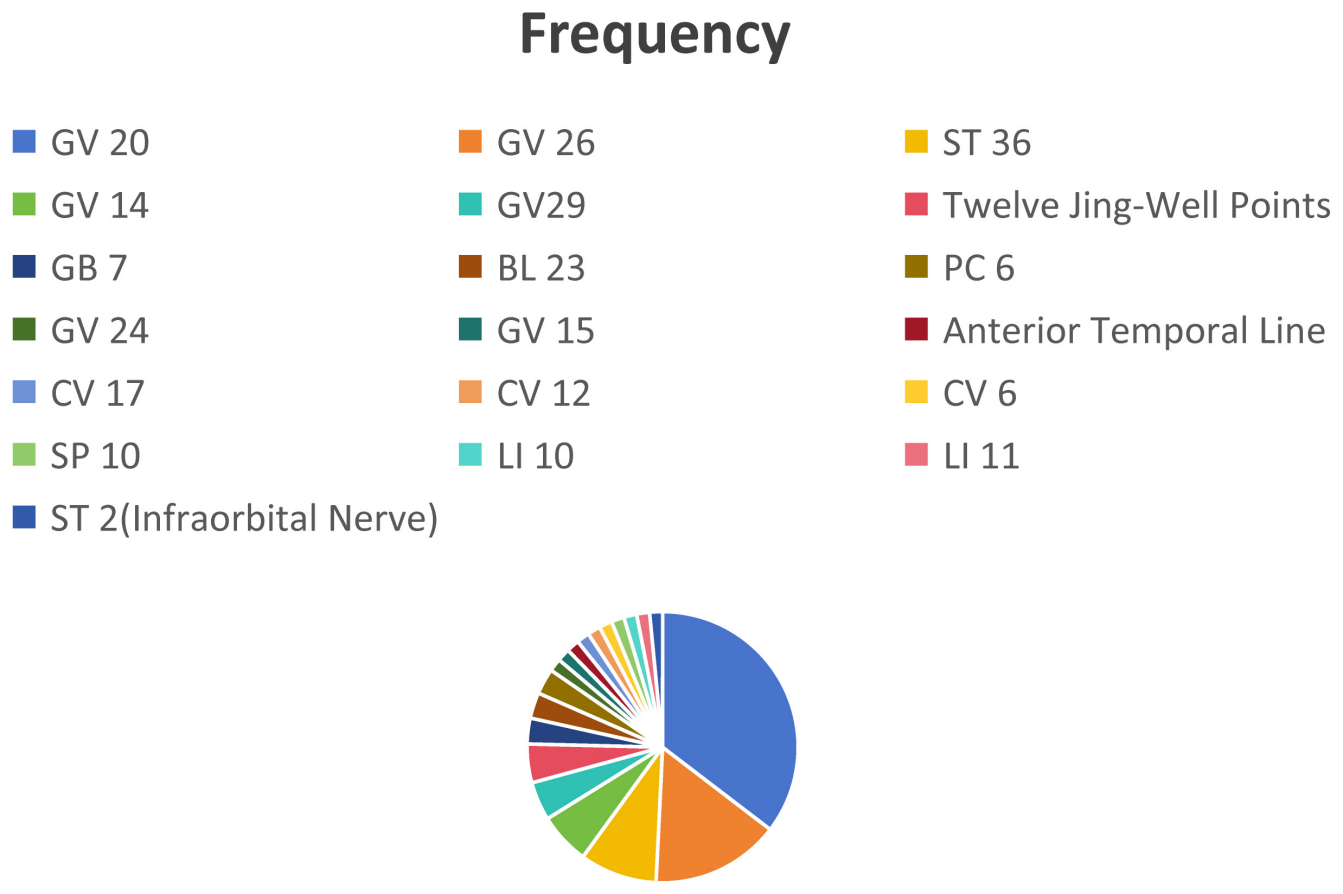
Statistical analysis of acupuncture point selection.

This study included 32 controlled trials, encompassing 424 rodent models rats/mice (212 animals in the intervention group and 212 in the comparison group). The acupuncture interventions contained electroacupuncture (15, 19–29, 32, 33, 35–37, 39, 41–43, 45, 46) (accounting for 72% of the studies), manual acupuncture (11, 12, 34, 38, 40), bloodletting acupuncture (14, 30, 31), and moxibustion (44) (together comprising 28%). In the electroacupuncture studies, 9 studies (15, 20, 23, 24, 26, 37, 39, 41, 45) used a continuous wave mode, and 14 studies (19, 21, 22, 25, 27–29, 32, 33, 35, 36, 42, 43, 46) used a sparse-dense wave mode. Three studies (22, 29, 32) applied an additional 6-s stimulation/6-s interval cycle on the sparse-dense wave. Manual acupuncture primarily used low-frequency stimulation (60–210 rotations per minute) (12, 34, 38, 40), mostly in an intermittent stimulation mode. Bloodletting at the twelve well-points was controlled at a dose of 15–20 μL per acupoint (14, 30). Only one study included moxibustion (44), which used mild moxibustion for continuous stimulation. The study characteristic table (Table 2; Supplementary Table 4) indicates that the selection of acupoints, stimulation parameters, intervention duration, and treatment frequency are also factors influencing the final effect size. Figure 2 further illustrates the frequency distribution of acupoint usage.

**Table 2:** Summary of study characteristics.

**Study**	**Animal model**	**Acupoints**	**Acupuncture protocol**	**Outcome**
Deng et al. (19)	SD, VD	GV20, GV14, BL23	EA 6/w, 4w	EB; IL-1β
Dong et al. (20)	SD, MCAO	GV20	EA 1/d, 5d	EB; MMP-9
Fan et al. (21)	SD, BI	GV20, GV26	EA 1/d, 1d	EB; MMP-9 mRNA; IL-1β, TNF-α & IL-6mRNA
Gong et al. (22)	SD, Aging rats	GV26, ST2	EA 1/d, 1d	EB; Occludin
He et al. (23)	SD, MCAO	GV20, ST36	EA 6/w, 8w	ZO-1; Iba-1; IL-1β, TNF-α, IL-6
Jung et al. (24)	C57 *BL/6J*, MCAO	GV20, GV14	EA 1/d, 3d	EB; occludin, claudin-5, ZO-1; GFAP
Lang et al. (25)	SD, SAH	GV20, GV14	EA 1/d, 3d	EB; occludin, claudin-5; MMP-9; Iba-1;IL-1β, TNF-α, IL-6
Li et al. (26)	SD, ICH	GV20, GB7	EA 1/d, 8d	EB
Lin et al. (27)	SD, MCAO	GV20, GV24	EA 1/d, 7d	MMP-9
Lin et al. (28)	SD, MCAO	GV20, GV26	EA 1/d, 15d	MMP-9
Lin et al. (29)	SD, MCAO	GV20, GV26	EA 1/d, 1d	EB; Occludin, ZO-1
Liu et al. (30)	SD, TBI	Twelve Jing-Well Points	BL 2/d, 2d	EB; MMP9; AQP4
Lu et al. (31)	Wistar, MCAO	Twelve Jing-Well Points	BL 2/d, 3d	EB
Ma et al. (32)	SD, PT	GV20, GV26	EA 1/d, 1d	EB; Occludin
Peng et al. (33)	SD, MCAO	GV20, GV26	EA 1/d, 1d	AQP4
Wang et al. (34)	*SAMP8*,AD	CV17, CV12, CV6, ST36, SP10	MA 6/w, 4w	EB; Occludin, claudin-5, ZO-1
Wang et al. (35)	SAMP8,AD	GV29, LI20	EA 5/w, 4w	claudin-5, ZO-1; Iba-1; IL-1β, TNF-α, IL-6
Wu et al. (36)	SD, MCAO	GV20, GV26	EA 1/d, 1d	EB
Xin et al. (37)	SD, SAE	GV20, ST36	EA 1/d, 4d	Occludin, ZO-1; Iba-1, GFAP; IL-6, IL-1β, TNF-α
Xu et al. (15)	SD, CIRI	GV20, ST36	EA 1/d, 2d	AQP4
Yao et al. (38)	SD, MCAO	Anterior Tem-poral Line	MA 1/d, 6d	EB; Occludin & ZO-1 mRNA; IL-1β
Yu et al. (39)	KM, Normal	GV20, GV15	EA 1/d, 1d	EB
Yu et al. (14)	Wistar, pMCAO	Twelve Jing-Well Points	BL 1/d, 4d	EB; Occludin & claudin-5 mRNA
Zhang et al. (40)	SD, ICH	GV20, GB7	MA 1/d, 7d	EB; Occludin
Zhang et al. (41)	SD, MCAO	GV20, GV26	EA 1/d, 1d	EB
Zhang et al. (42)	SD, Nomal	GV20, GV26	EA 1/d, 1d	EB; Occludin, claudin-5, ZO-1; Iba1, GFAP; AQP4
Zhang et al. (43)	SD, MCAO	GV26, PC6	EA 1/d, 1d	EB; claudin-5, ZO-1; MMP-9
Zhang et al. (11)	*APP/PS1*, AD	GV20, GV29, ST36	MA 1/d, 38d	EB; Occludin, ZO-1
Zhang et al. (12)	SD, MCAO	GV20, PC6	MA 1/d, 1d	EB; Occludin, ZO-1
Zhou et al. (44)	SD, AD	GV20, BL23, GV29	Mox 1/d, 21d	EB; MMP-9
Zhu et al. (45)	*APP/PS1*, POCD	GV20, GV14, ST36, LI11	EA 1/d, 9d	Occludin;Iba-1, GFAP; IL-1β, TNF-α, IL-6
Zou et al. (46)	SD, MCAO	GV20	EA 1/d, 5d	Occludin, claudin-5

VD, Vascular Dementia; MCAO, Middle Cerebral Artery Occlusion Model; BI, Brain X-ray Radiation; SAH, Subarachnoid Hemorrhage; ICH, Spontaneous Intracerebral Hemorrhage; TBI, Traumatic Brain Injury; PT, C6 Glioma Model; AD, Alzheimer's Disease; SAE, Sepsis-Associated Encephalopathy; CIRI, Cerebral Ischemia/Reperfusion Injury; pMCAO, Permanent Middle Cerebral Artery Occlusion; POCD, Postoperative Cognitive Dysfunction; EA, Electroacupuncture; MA, Manual acupuncture; Mox, moxibustion; KM, Kun ming mice; BL, Bloodletting; NR, No mention was made of this in the study.

#### 3.2.2 Selection of outcome target molecules

**Figure 3:**
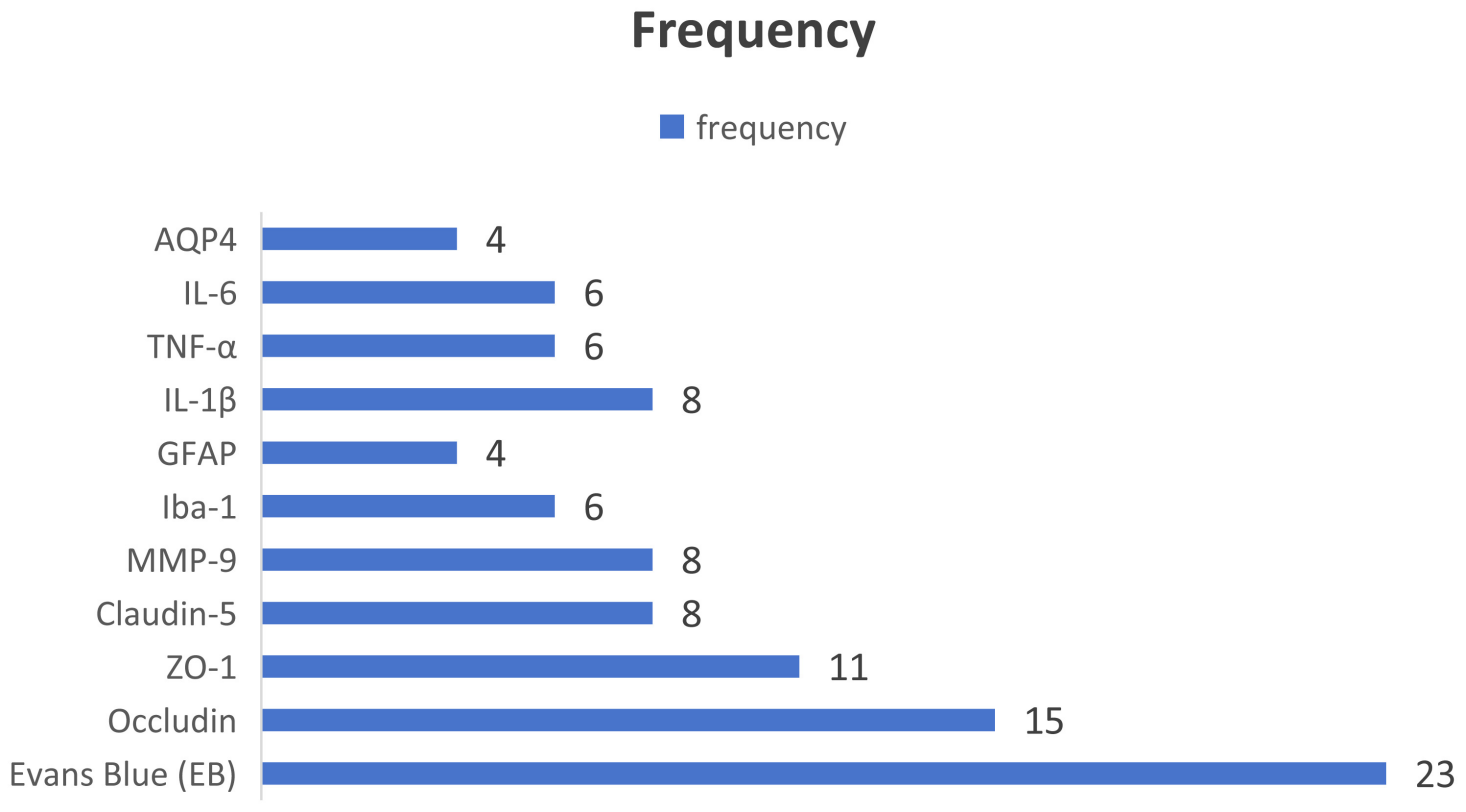
Frequency-based selection of outcome target molecules (threshold: ≥4 studies).

We identified the outcome target molecules through a frequency analysis, defining inclusion as the molecule being reported in a minimum of four studies (Figure 3).

### 3.3 Quality evaluation

**Figure 4:**
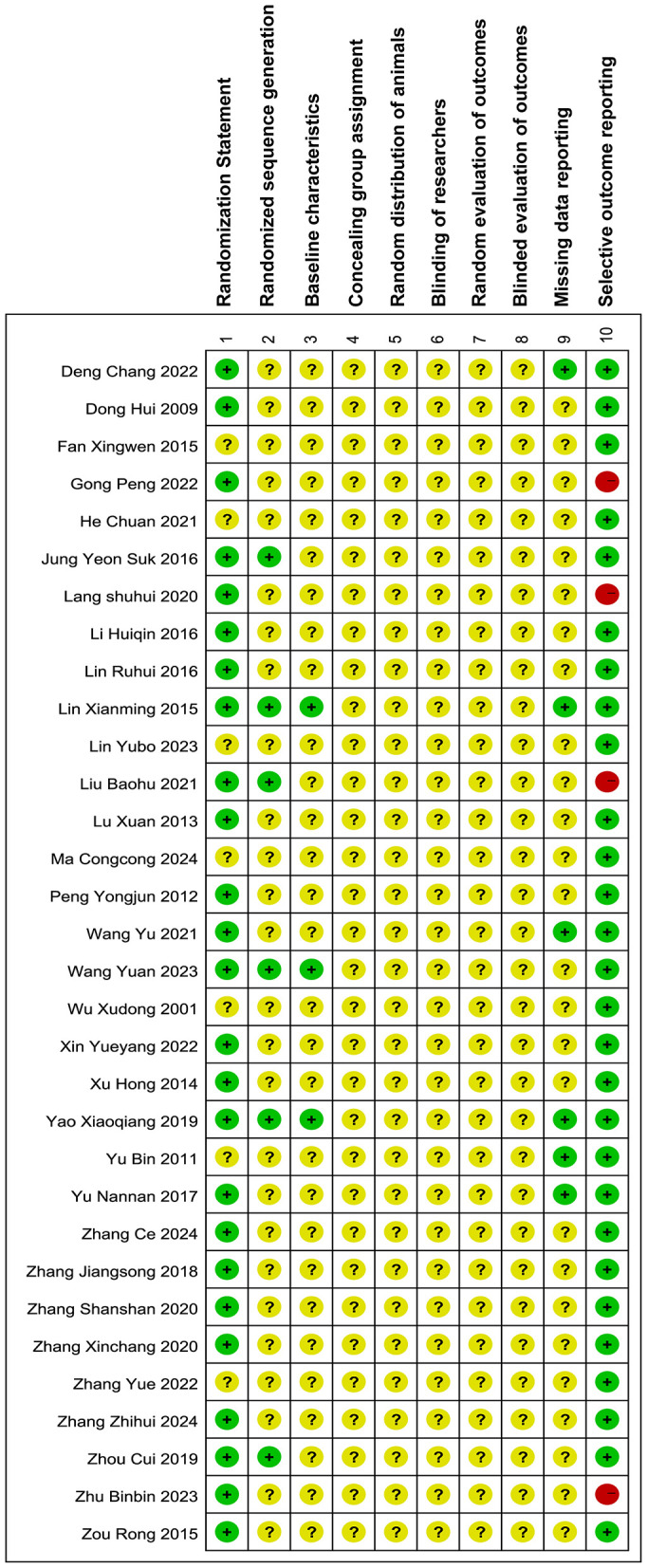
Bias risk of the included researches. The assessment of the 32 papers was carried out with RevMan 5.4.1 along with SYRCLE's quality assessment tool. An additional point regarding the description of randomization methods (Item 1) was included, while the “Additional sources of bias” section was excluded from this review. Notations utilized: 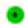: low risk; 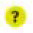:unclear risk; 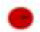: high risk.

Thirty two articles were included in the quality assessment, as shown in Figure 4. Seventy eight percent of the articles (12, 14, 15, 19, 20, 22, 24–28, 30, 31, 33–35, 37, 38, 40–46) had a randomization statement, but only 19% explicitly described the randomization method (24, 28, 30, 35, 38, 44). Thirteen percent of the included studies (22, 25, 30, 45) had a high risk of selective reporting. No studies explicitly described allocation concealment, randomization of animal placement, random outcome assessment, blinded outcome evaluation, or blinding of the experimenters.

### 3.4 Meta-analysis results

#### 3.4.1 BBB permeability and tight junction proteins

##### 3.4.1.1 BBB permeability

**Figure 5:**
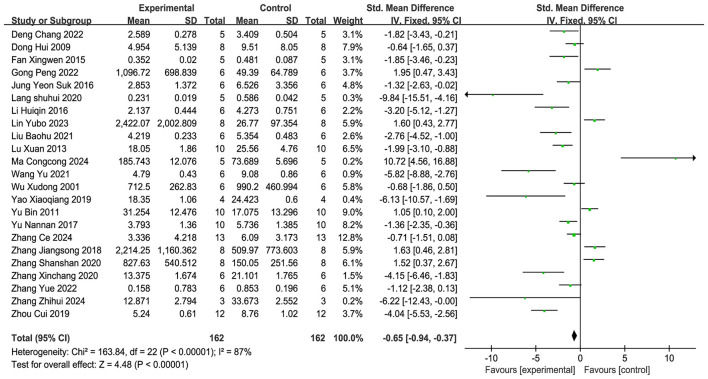
Forest plot of EB extravasation.

A total of 23 studies (11, 12, 14, 19–22, 24–26, 29–32, 34, 36, 38–44) were comprised in the analysis. and the meta-analysis (Figure 5) demonstrated that, in comparison with the control group, acupuncture significantly reduced Evans Blue (EB) extravasation in brain tissue (SMD = −0.65, 95% CI: −0.94 to −0.37, *P* < 0.01), indicating that acupuncture can inhibit EB leakage into the brain tissue across the BBB and reduce BBB permeability. Funnel plot analysis (Supplementary Figure 1A) was used to visually assess potential publication bias, showing a roughly symmetric distribution of scatter points, suggesting no significant asymmetry. Further quantitative verification was conducted using the Egger linear regression test, and the findings indicated that the test statistic *P* = 0.053 > 0.05, displaying no statistically significant publication bias. To assess the reliability of the findings, sensitivity analysis was conducted by sequentially removing individual studies. The results (Supplementary Figure 1B) showed minimal fluctuation in the combined effect size (SMD = −2.34 to −0.51). This indicates that the conclusions of the current meta-analysis have low dependence on individual studies, demonstrating high robustness, with conclusions not significantly affected by outliers or bias from individual studies.

##### 3.4.1.2 Tight junction proteins

**(1) Occludin**.

**Figure 6:**
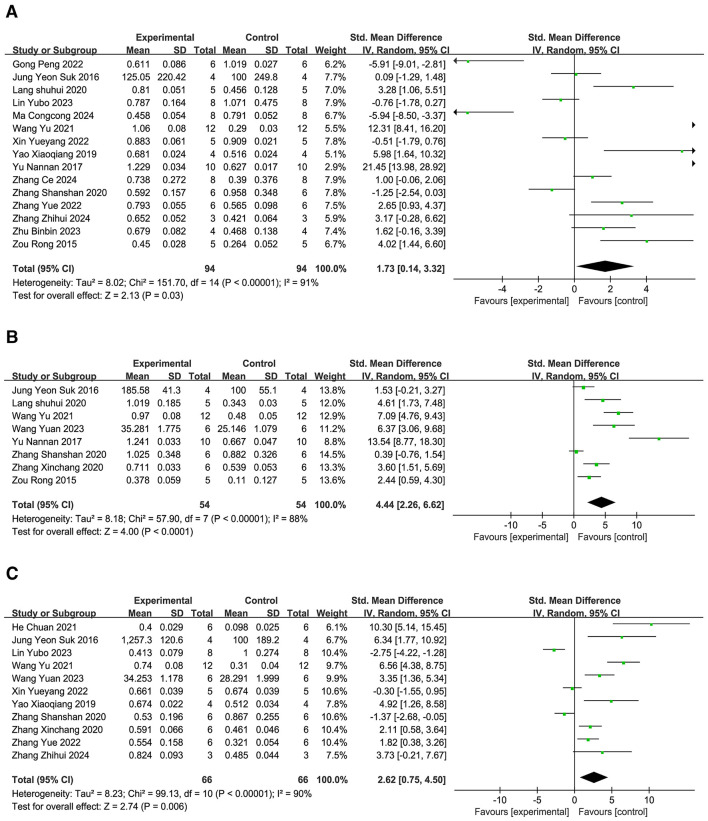
Forest plot of tight junction protein expression. **(A)** Occludin. **(B)** Claudin-5. **(C)** ZO-1.

A total of 15 studies (11, 12, 14, 22, 24, 25, 29, 32, 34, 37, 38, 40, 42, 45, 46) were comprised in the examination, and the meta-analysis (Figure 6) demonstrated that, relative to the comparison group, the expression of occludin protein and mRNA in brain tissue was significantly upregulated by acupuncture (SMD = 1.73, 95% CI: 0.14–3.32, *P* < 0.01).The findings point out that acupuncture could enhance occludin expression in the cerebral tissue, potentially strengthening BBB integrity. Funnel plot analysis (Supplementary Figure 2A) showed a roughly symmetric distribution of scatter points, and Egger linear regression test results (*P* = 0.059 > 0.05) confirmed no major publication bias. Sensitivity evaluation revealed that the combined effect size range remained stable (SMD = 0.30–3.70, Supplementary Figure 3A), suggesting robust results, with conclusions not significantly dependent on individual studies.

**(2) Claudin-5**.

Eight studies (14, 24, 25, 34, 35, 42, 43, 46) were included, and the meta-analysis (Figure 6) demonstrated that, in comparison to the reference group, acupuncture substantially promoted the production of claudin-5 protein and mRNA in brain tissue (SMD = 4.44, 95% CI: 2.26–6.62, *P* < 0.01). This result suggests acupuncture may enhance claudin-5 levels in brain tissue. Sensitivity analysis, conducted by sequentially removing individual studies (Supplementary Figure 3B), showed that the range of the combined effect size remained stable (SMD = 2.54–7.07), indicating robust results with conclusions not significantly dependent on individual studies.

**(3) ZO-1**.

The assessment included eleven studies (11, 12, 23, 24, 29, 34, 35, 37, 38, 42, 43), and the meta-analysis (Figure 6) implied that, as opposed to the comparison group, acupuncture significantly increased the level of ZO-1 protein and mRNA in brain tissue (SMD = 2.62, 95% CI: 0.75–4.5, *P* < 0.01). This indicates that acupuncture could elevate ZO-1 expression. The Egger linear regression analysis revealed a notable publication bias (*P* = 0.01 < 0.05). To correct for potential bias, the data were adjusted using the trim-and-fill method. After supplementing two imputed missing studies, the updated funnel plot (Supplementary Figure 2B) showed that the effect size points of each study tended to be symmetrically distributed on both sides, indicating partial correction of publication bias. Sensitivity analysis (Supplementary Figure 3C) indicated that the combined effect size range remained stable (SMD = 1.01–5.04), demonstrating robust results, with conclusions not significantly dependent on individual studies.

#### 3.4.2 Matrix metalloproteinase

**Figure 7:**
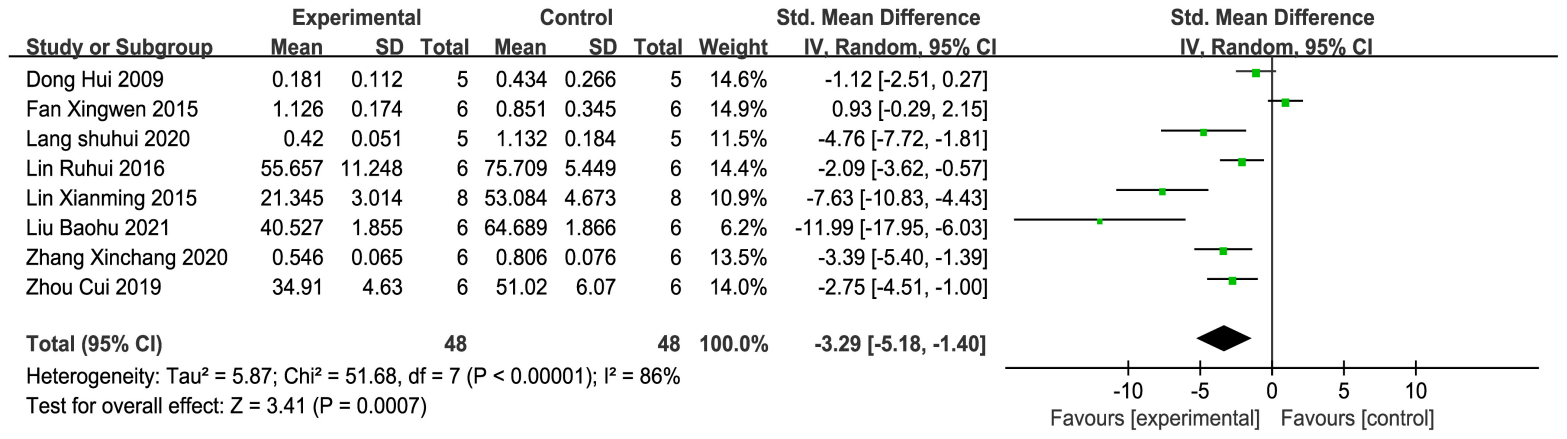
Forest plot of MMP-9 expression.

The analysis comprised eight studies (20, 21, 25, 27, 28, 30, 43, 44), and the meta-analysis (Figure 7) revealed that, in contrast to the comparison group, acupuncture markedly decreased the MMP-9 protein and mRNA levels in brain tissue (SMD = −3.29, 95% CI: −5.18 to −1.40, *P* < 0.001). This points to the possibility that acupuncture could reduce the expression of MMP-9. Sensitivity analysis applying the leave-one-out approach (Supplementary Figure 4) revealed that the combined effect size range remained stable (SMD = −5.70 to −1.63), indicating the robustness of the results and that the conclusions are not significantly dependent on individual studies.

#### 3.4.3 Glial activation markers and inflammatory mediators

##### 3.4.3.1 Glial activation markers

**(1) Iba-1**.

**Figure 8:**
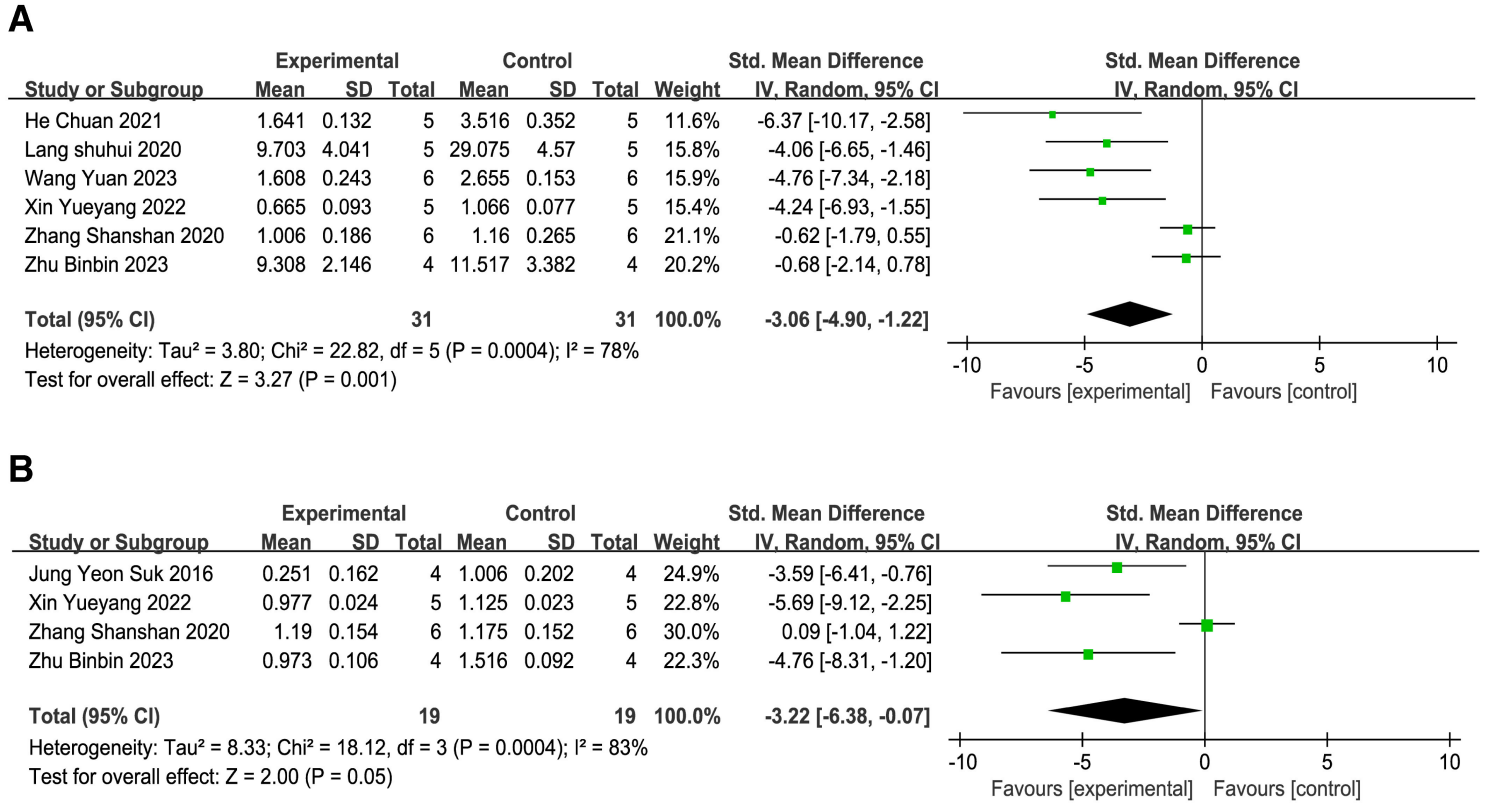
Forest plot of the expression of glial activation markers. **(A)** Iba-1. **(B)** GFAP.

Six studies (23, 25, 35, 37, 42, 45) incorporated into the evaluation. The evaluation (Figure 8) revealed that, relative to the comparison group, acupuncture significantly inhibited the production of Iba-1 in brain tissue (SMD = −3.06, 95% CI: −4.90 to −1.22, *P* < 0.001). This indicates that acupuncture has the potential to effectively suppress the activation of microglial cells. Sensitivity analysis with the leave-one-out procedure (Supplementary Figure 5A) revealed that the combined effect size range remained stable (SMD = −5.48 to −1.45), indicating the robustness of the results and that the conclusions are not significantly dependent on individual studies.

**(2) GFAP**.

Four studies (24, 37, 42, 45) were incorporated into the examination, and the meta-analysis (Figure 8) demonstrated that, relative to the reference group, acupuncture significantly inhibited the production of GFAP in brain tissue (SMD = −3.22, 95% CI: −6.38 to −0.07, *P* < 0.001). This indicates that acupuncture can effectively suppress the activation of astrocytes. Sensitivity analysis utilizing the leave-one-out strategy (Supplementary Figure 5B) revealed that the combined effect size range remained stable (SMD = −7.27 to −0.21), indicating the robustness of the results and that the conclusions are not significantly dependent on individual studies.

##### 3.4.3.2 Inflammatory mediators

**(1) IL-1β**.

**Figure 9:**
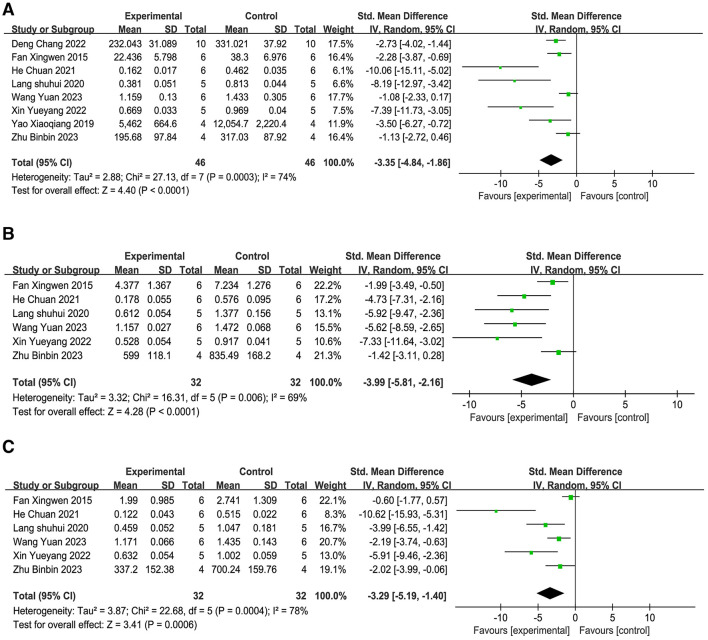
Forest plot of the expression of inflammatory mediators. **(A)** IL-1β. **(B)** TNF-α. **(C)** IL-6.

The assessment comprised eight studies (19, 21, 23, 25, 35, 37, 38, 45), and the meta-analysis (Figure 9) revealed that, as opposed to the comparison group, acupuncture significantly inhibited the IL-1β protein and mRNA amounts in brain tissue (SMD = −3.35, 95% CI: −4.84 to −1.86, *P* < 0.001). This points to the possibility that acupuncture could suppress the production of IL-1β in brain tissue. Sensitivity analysis based on the leave-one-out method technique (Supplementary Figure 6A) demonstrated that the combined effect size range remained stable (SMD = −5.52 to −2.24), indicating the robustness of the results and that the conclusions are not significantly dependent on individual studies.

**(2) TNF-α**.

The assessment comprised six studies (21, 23, 25, 35, 37, 45), and the meta-analysis (Figure 9) confirmed that, as opposed to the comparison group, acupuncture significantly inhibited the amounts of TNF-α protein and mRNA in brain tissue (SMD = −4.41, 95% CI: −7.07 to −1.74, *P* < 0.001). This Indicates that acupuncture could suppress the release of TNF-α in brain tissue. Sensitivity analysis employing the leave-one-out technique (Supplementary Figure 6B) showed that the combined effect size range remained stable (SMD = −7.77 to −2.04), indicating the robustness of the results and that the conclusions are not significantly dependent on individual studies.

**(3) IL-6**.

Six studies (21, 23, 25, 35, 37, 45) incorporated into the final evaluation, and the meta-analysis (Figure 9) showed that, as opposed to the reference group, the acupuncture intervention led to a significant reduction in the protein and mRNA levels of IL-6 in brain tissue. (SMD = −3.29, 95% CI: −5.19 to −1.40, *P* < 0.001). This implies that acupuncture may inhibit the production of IL-6 within brain tissue. Sensitivity analysis applying the leave-one-out approach (Supplementary Figure 6C) showed that the combined effect size range remained stable (SMD = −5.85 to −1.71), indicating that the results are robust and that the conclusions are not significantly dependent on individual studies.

#### 3.4.4 Water channel protein

**Figure 10:**
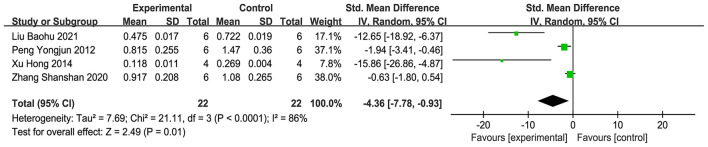
Forest plot of AQP4 expression.

Four studies (15, 30, 33, 42) were contained in the assessment, and the meta-analysis (Figure 10) showed that, in contrast to the comparison group, the acupuncture group notably diminished the AQP4 protein and mRNA levels in brain tissue (SMD = −4.36, 95% CI: −7.78 to −0.93, *P* < 0.001). This suggests that acupuncture may inhibit the level of AQP4 in brain tissue. Sensitivity analysis with the leave-one-out procedure (Supplementary Figure 7) showed that the combined effect size range was unstable (SMD = −9.23 to −1.58).

## 4 Discussion

This study, through the preclinical Meta-analysis, integrated preclinical data on acupuncture's influence regarding BBB permeability. The findings indicate that acupuncture treatment notably decreased EB leakage in brain tissue, upregulated the production of tight junction proteins (occludin, claudin-5, ZO-1), and simultaneously inhibited the expression of matrix metalloproteinase-9 (MMP-9), glial cell activation markers (Iba-1, GFAP), inflammatory mediators (IL-1β, IL-6, TNF-α), and aquaporin-4 (AQP4). These findings indicate that acupuncture might improve BBB integrity with a multi-target synergistic effect.

### 4.1 Acupuncture's regulation of BBB structure and tight junction proteins

Our research results show that acupuncture intervention can decrease EB extravasation in brain tissue and upregulate the synthesis of tight junction proteins (occludin, claudin-5, ZO-1). EB is a commonly used marker to evaluate BBB permeability. The reduction in EB levels in the acupuncture intervention group directly indicates that acupuncture can reduce the dye's entry into the brain tissue, reflecting that acupuncture may decrease BBB permeability, thereby preventing large molecules from entering the brain from the bloodstream. The protective role of the BBB depends on the integrity of tight junctions (TJs) between endothelial cells, with occludin, claudin-5, and ZO-1 being the core components of TJs (8). Therefore, occludin and claudin-5 may enhance the mechanical strength of endothelial cell junctions upon upregulation, while ZO-1, as a key anchoring molecule that connects tight junction (TJ) proteins to the actin cytoskeleton, may further stabilize the spatial conformation of the TJs complex (47). The lack of ZO-1 results in the disruption of the BBB in many neurodegenerative and acute CNS diseases (8). The regulation of TJs by acupuncture suggests that it may influence endothelial cell function through pathways such as RhoA/ROCK II/MLC 2 (40), Wnt/β-catenin (48, 49) and HIF-1α (50, 51).

### 4.2 MMP-9 inhibition and extracellular matrix protection

Our research results show that acupuncture intervention can inhibit the amount of matrix MMP-9 expression. MMP-9 is primarily secreted by glial types like astrocytes, microglia and endothelial cells within the central nervous system (52). It can disrupt the BBB structure and exacerbate BBB leakage by degrading the extracellular matrix (ECM) and TJ proteins (52, 53). The reduction in MMP-9 expression by acupuncture may be achieved through two main mechanisms as follows. Firstly inhibition of NF-κB or MAPK signaling pathways, which reduces the transcriptional activation of MMP-9 (30, 43, 54–56). Secondly upregulation of tissue inhibitors of metalloproteinases (TIMPs), restoring the MMP-9/TIMP-1 balance (57, 58). For example, Acupuncture bloodletting at the twelve Jing-well acupoints on the hands, reduces BBB permeability, and downregulates MMP9 expression in rats suffering from severe traumatic brain injury by inhibiting the MAPK signaling pathway (30). The suppression of MMP-9 not only protects the structure of TJs but may also reduce inflammatory cell infiltration, forming a positive feedback loop (25, 59).

### 4.3 Synergistic inhibition of glial cell activation and inflammatory response

Our research findings demonstrate that acupuncture intervention can inhibit the amount of astrocyte activation markers (GFAP), microglia activation markers (Iba-1), pro-inflammatory factors (IL-1β, IL-6, TNF-α), and aquaporin-4 (AQP4). The excessive activation of microglia (Iba-1+) and astrocytes (GFAP+) is a key driver of neuroinflammation and Blood–Brain barrier (BBB) disruption (60, 61). Acupuncture significantly reduces Iba-1 and GFAP protein levels, demonstrating a suppressive effect on neuroinflammatory glial responses. The suppression of glial activation may reduce BBB damage through two main mechanisms as follows. First, decreasing the secretion of pro-inflammatory substances (TNF-α, IL-6, IL-1β), blocking their destructive effects on endothelial tight junctions (25, 35, 45, 62). Second, downregulating the production of harmful agents like reactive oxygen species (ROS) and nitric oxide (NO), alleviating oxidative stress (21, 24, 35, 63). Meanwhile, inhibiting the generation of inflammatory molecules like TNF-α and IL-1β can alleviate glial cell activation (37), forming a negative feedback loop, thereby reducing NF-κB pathway activation and ultimately improving Blood–Brain barrier permeability (23, 64). Moreover, the reduction in AQP4 expression may be associated with the alleviation of astrocytic end-foot swelling, which reduces the mechanical pressure of vasogenic edema on the BBB, further maintaining barrier stability (65).

### 4.4 Potential association between AQP4 downregulation and cerebral edema

Our research results show that acupuncture intervention can reduce the protein and mRNA levels of AQP4. AQP4, as the principal water transport protein in astrocytic end-feet, is closely associated with increased BBB permeability and brain edema when its expression is abnormal (66, 67). Acupuncture reduces AQP4 protein and mRNA levels, potentially through two main mechanisms as follows. Firstly, inhibiting the transcriptional regulation of AQP4 by inflammatory signals (30) (e.g., MAPK). Secondly, reducing the disruption of AQP4 polarity distribution caused by glial cell activation (68). The downregulation of AQP4 may limit the transmembrane movement of water molecules, thereby alleviating vasogenic or cytotoxic edema, and working synergistically with tight junction repair to maintain BBB homeostasis (24).

The above findings indicate that acupuncture's protective role in the BBB may involve a multi-target regulatory mechanism. It achieves this by coordinating the interactions between endothelial cells, glial cells (69), and immune signaling molecules, thereby promoting the functional coordination and homeostatic reconstruction of various cellular components within the neurovascular unit (NVU). For example, inhibiting MMP-9 reduces the degradation of tight junctions (TJs), while decreased levels in IL-6 and TNF-α may inhibit glial cell activation via the NF-KB pathway. Additionally, the downregulation of AQP4 further blocks the vicious cycle of edema and inflammation (70).

## 5 Limitations and future perspectives

The limitations of this study are as follows: (1) The impact of acupuncture on the BBB in both physiological and pathological conditions, as well as its effects during the acute and recovery phases of disease, were not explored; (2) The study included four types of acupuncture—electroacupuncture, manual acupuncture, bloodletting, and moxi-bustion—but did not investigate whether these different acupuncture methods vary in their effects on the BBB or their effect sizes; (3) The influence of acupuncture parameters, such as acupoint, frequency, and intervention duration, on effect size was not further analyzed; (4) The small number of included studies and limited sample size might result in an overestimation of effect size; (5) The execution of randomization, allocation concealment, and blinding in most studies was unclear, which may also result in an overestimation of effect size; (6) Some outcome measures were assessed using different methods, which may introduce heterogeneity in measurement techniques, such as using different antibodies to detect Occludin; (7) The pathological process in animal models (e.g., MCAO stroke model) may differ from the natural progression of human disease, making it difficult to fully extrapolate the preclinical evidence on acupuncture's effect on BBB permeability to clinical settings. Additional clinical evidence is required to assess if acupuncture is a viable option in clinical practice.

In future research, we anticipate further exploration in the following areas: (1) Investigating the impact of acupuncture upon the BBB in various physiological and pathological states, particularly during the acute and recovery phases of disease. Comparative studies of disease models at these different stages will help elucidate the healing effects and pathways of acupuncture throughout various stages of disease progression; (2) Examining the differences in the effects of various acupuncture interventions—such as electroacupuncture, manual acupuncture, bloodletting, and moxibustion—on BBB permeability, as well as their respective indications. This will provide clearer guidance for clinical treatment; (3) Deepening the study of acupuncture parameter optimization, with a focus on key parameters such as acupoint combinations (single point or multiple points), stimulation frequency, and intervention timing (prevention, treatment, or rehabilitation), to understand their effects on BBB regulation and provide data for clinical translation.

## 6 Conclusion

In summary, acupuncture may improve BBB integrity by means of multiple mechanisms, including the enhancement of tight junction protein production in endothelial cells, inhibiting MMP-9 mediated extracellular matrix (ECM) degradation, modulating glial cell activation and inflammatory responses, and downregulating AQP4-dependent edema. These results establish a data foundation for acupuncture in the management of neurological illnesses like stroke and Alzheimer's disease, emphasizing the importance of an integrated treatment strategy targeting the neurovascular unit (NVU).

## Data Availability

Publicly available datasets were analyzed in this study. This data can be found here: The data in this study were extracted from 32 included studies, and the specific sources are listed in the references section of the main text.
