# Supplementary material for: Acupuncture improves blood–brain barrier integrity through multi-targeted mechanisms: a preclinical meta-analysis
Source: Front Neurol. 2025 Nov 7;16:1648117. doi: 10.3389/fneur.2025.1648117 (PMC12636094; doi:10.3389/fneur.2025.1648117)
Supplement: Supplementary file 1 [file Supplementary_Table_1.docx]

**Supplementary Table 1.** Search strategy on embase.

| #1 | 'acupuncture'/exp OR acupuncture OR pharmacopuncture OR (acupuncture AND therapy) OR (acupuncture AND treatment) OR (acupuncture AND treatments) OR (treatment, AND acupuncture) OR (therapy, AND acupuncture) OR (pharmacoacupuncture AND treatment) OR (treatment, AND pharmacoacupuncture) OR (pharmacoacupuncture AND therapy) OR (therapy, AND pharmacoacupuncture) OR acupotomy OR acupotomies OR (acupuncture, AND ear) OR (acupunctures, AND ear) OR (ear AND acupunctures) OR (acupuncture, AND auricular) OR (acupunctures, AND auricular) OR (auricular AND acupunctures) OR (auricular AND acupuncture) OR (ear AND acupuncture) OR (acupuncture AND points) OR (acupuncture AND point) OR (point, AND acupuncture) OR (points, AND acupuncture) OR acupoints OR acupoint OR (analgesia, AND acupuncture) OR (acupuncture AND analgesia) OR (anesthesia, AND acupuncture) OR (acupuncture AND anesthesia) |
| --- | --- |
| #2 | 'blood-brain barrier'/exp OR 'blood-brain barrier' OR ('blood brain' AND ('barrier'/exp OR barrier)) OR (barrier, AND 'blood brain') OR (barriers, AND 'blood brain') OR (blood AND brain AND barrier) OR ('blood brain' AND barriers) OR ('hemato encephalic' AND barrier) OR (barrier, AND 'hemato encephalic') OR (barriers, AND 'hemato encephalic') OR (hemato AND encephalic AND barrier) OR ('hemato encephalic' AND barriers) OR ('brain blood' AND barrier) OR (barrier, AND 'brain blood') OR (barriers, AND 'brain blood') OR (brain AND blood AND barrier) OR ('brain blood' AND barriers) |
| #3 | (#1) OR (#2) |
