# Supplementary material for: Acupuncture improves blood–brain barrier integrity through multi-targeted mechanisms: a preclinical meta-analysis
Source: Front Neurol. 2025 Nov 7;16:1648117. doi: 10.3389/fneur.2025.1648117 (PMC12636094; doi:10.3389/fneur.2025.1648117)
Supplement: Supplementary file 2 [file Supplementary_Table_2.docx]

**Supplementary Table 2.** Search strategy on Cochrane Library.

| #1 | (Acupuncture) OR (Acupuncture Therapy) OR (Acupuncture, Ear) OR (Acupuncture Points) OR (Acupuncture Analgesia) |
| --- | --- |
| #2 | (Acupuncture Treatment) OR (Acupuncture Treatments) OR (Treatment, Acupuncture) OR (Therapy, Acupuncture) OR (Pharmacoacupuncture Treatment) |
| #3 | (Treatment, Pharmacoacupuncture) OR (Pharmacoacupuncture Therapy) OR (Therapy, Pharmacoacupuncture) OR (Acupotomy) OR (Acupotomies) |
| #4 | (Acupunctures, Ear) OR (Ear Acupunctures) OR (Acupuncture, Auricular) OR (Acupunctures, Auricular) OR (Auricular Acupunctures) |
| #5 | (Acupuncture Point) OR (Point, Acupuncture) OR (Points, Acupuncture) OR (Acupoints) OR (Acupoint) |
| #6 | #1 OR #2 OR #3 OR #4 OR #5 |
| #7 | (Auricular Acupuncture) OR (Ear Acupuncture) OR (Analgesia, Acupuncture) OR (Acupuncture Anesthesia) OR (Anesthesia, Acupuncture) |
| #8 | (Pharmacopuncture) |
| #9 | #7 OR #8 OR #6 |
| #10 | (Barrier, Blood-Brain) OR (Barriers, Blood-Brain) OR (Blood Brain Barrier) OR (Blood-Brain Barriers) OR (Hemato-Encephalic Barrier) |
| #11 | (Barrier, Hemato-Encephalic) OR (Barriers, Hemato-Encephalic) OR (Hemato Encephalic Barrier) OR (Hemato-Encephalic Barriers) OR (Brain-Blood Barrier) |
| #12 | (Barrier, Brain-Blood) OR (Barriers, Brain-Blood) OR (Brain Blood Barrier) OR (Brain-Blood Barriers) OR (Blood-Brain Barrier) |
| #13 | #10 OR #11 OR #12 |
| #14 | #9 AND #13 |
