# Supplementary material for: Acupuncture improves blood–brain barrier integrity through multi-targeted mechanisms: a preclinical meta-analysis
Source: Front Neurol. 2025 Nov 7;16:1648117. doi: 10.3389/fneur.2025.1648117 (PMC12636094; doi:10.3389/fneur.2025.1648117)
Supplement: Supplementary file 3 [file Supplementary_Table_3.docx]

**Supplementary Table 3.** Search strategy on Web of Science.

| #1 | ((((((((((((((ALL=(Blood-Brain Barrier-mesh)) OR ALL=(Barrier, Blood-Brain)) OR ALL=(Barriers, Blood-Brain)) OR ALL=(Blood Brain Barrier)) OR ALL=(Blood-Brain Barriers)) OR ALL=(Hemato-Encephalic Barrier)) OR ALL=(Barrier, Hemato-Encephalic)) OR ALL=(Barriers, Hemato-Encephalic)) OR ALL=(Hemato Encephalic Barrier)) OR ALL=(Hemato-Encephalic Barriers)) OR ALL=(Brain-Blood Barrier)) OR ALL=(Barrier, Brain-Blood)) OR ALL=(Barriers, Brain-Blood)) OR ALL=(Brain Blood Barrier)) OR ALL=(Brain-Blood Barriers) |
| --- | --- |
| #2 | ((((((((((((((((((((((((((((((ALL=(Acupuncture)) OR ALL=(Pharmacopuncture)) OR ALL=(Acupuncture Therapy)) OR ALL=(Acupuncture Treatment)) OR ALL=(Acupuncture Treatments)) OR ALL=(Treatment, Acupuncture)) OR ALL=(Therapy, Acupuncture)) OR ALL=(Pharmacoacupuncture Treatment)) OR ALL=(Treatment, Pharmacoacupuncture)) OR ALL=(Pharmacoacupuncture Therapy)) OR ALL=(Therapy, Pharmacoacupuncture)) OR ALL=(Acupotomy)) OR ALL=(Acupotomies)) OR ALL=(Acupuncture, Ear)) OR ALL=(Acupunctures, Ear)) OR ALL=(Ear Acupunctures)) OR ALL=(Acupuncture, Auricular)) OR ALL=(Acupunctures, Auricular)) OR ALL=(Auricular Acupunctures)) OR ALL=(Auricular Acupuncture)) OR ALL=(Ear Acupuncture)) OR ALL=(Acupuncture Points)) OR ALL=(Acupuncture Point)) OR ALL=(Point, Acupuncture)) OR ALL=(Points, Acupuncture)) OR ALL=(Acupoints)) OR ALL=(Acupoint)) OR ALL=(Acupuncture Analgesia)) OR ALL=(Analgesia, Acupuncture)) OR ALL=(Acupuncture Anesthesia)) OR ALL=(Anesthesia, Acupuncture) |
| #3 | (#1) OR (#2) |
