# Supplementary material for: Acupuncture improves blood–brain barrier integrity through multi-targeted mechanisms: a preclinical meta-analysis
Source: Front Neurol. 2025 Nov 7;16:1648117. doi: 10.3389/fneur.2025.1648117 (PMC12636094; doi:10.3389/fneur.2025.1648117)
Supplement: Supplementary file 4 [file Data_Sheet_1.docx]

Supplementary Material

# 1 Supplementary Figures and Tables

- 1. **Supplementary Figures**

**
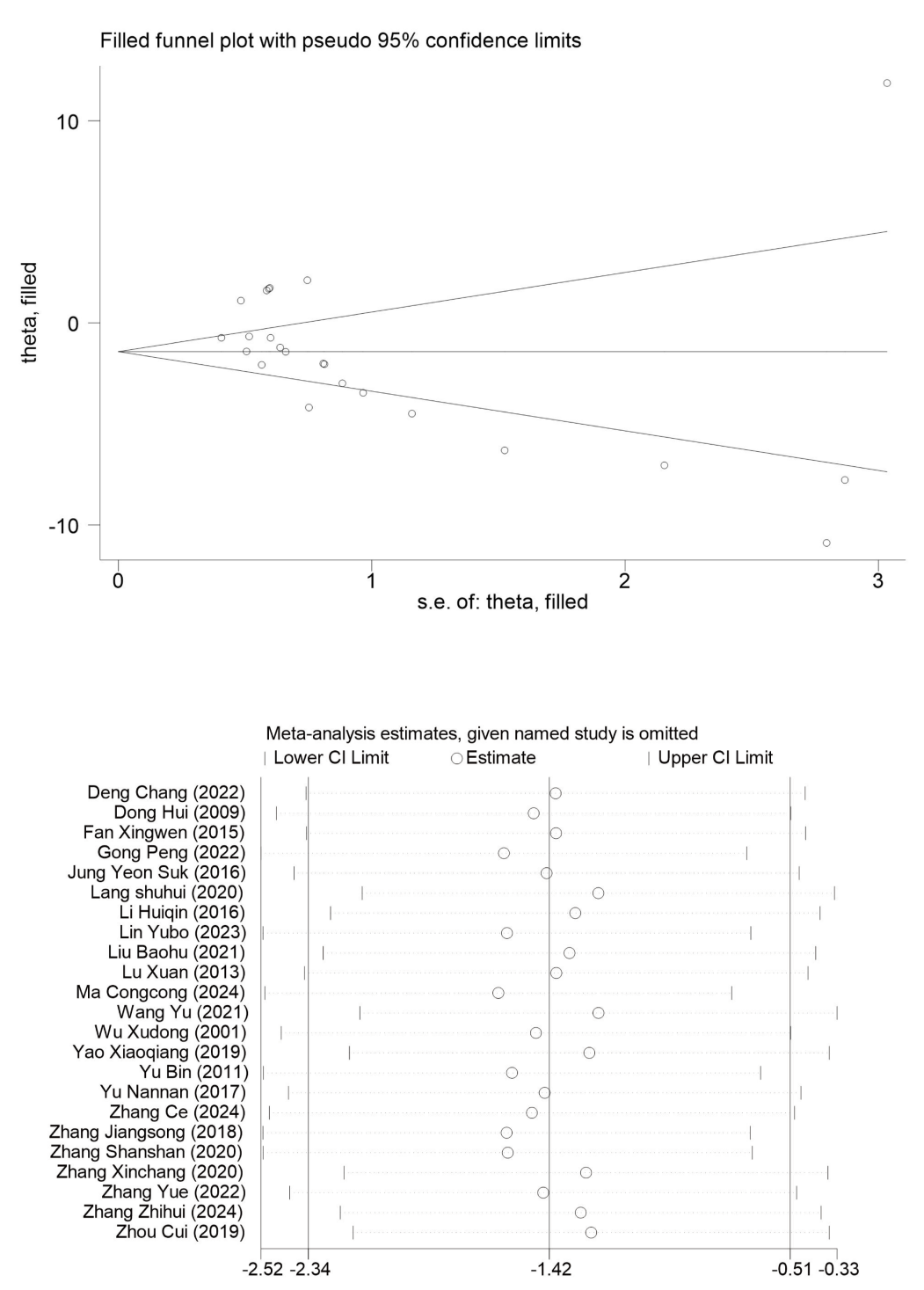
**

**Supplementary Figure 1.** Publication bias and sensitivity analysis for Evans Blue extravasation（EB）. (A) Funnel plot. (B) Sensitivity analysis.


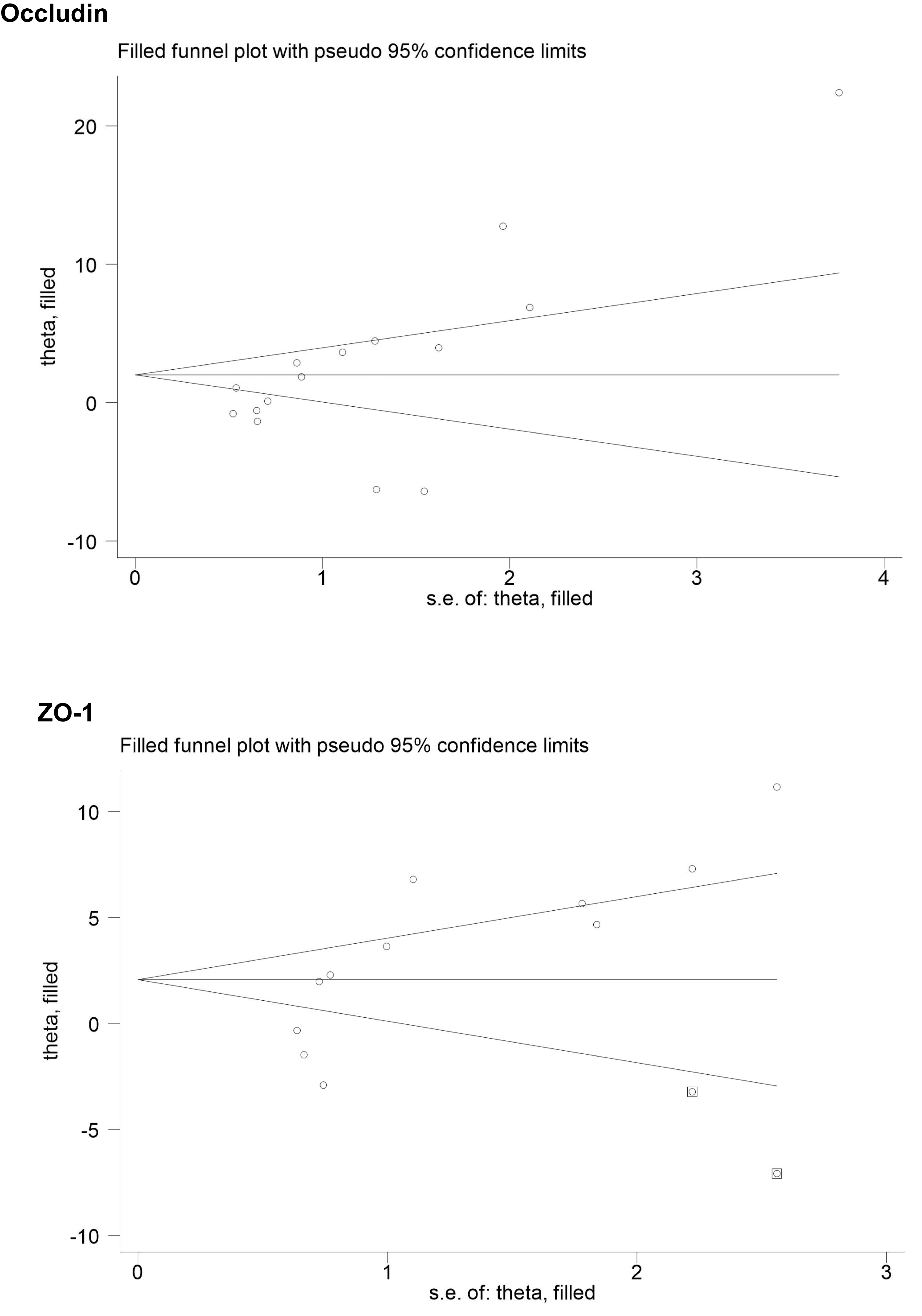


**Supplementary Figure 2.** Publication bias for the expression of tight junction proteins. (A) Occludin. (B) ZO-1.


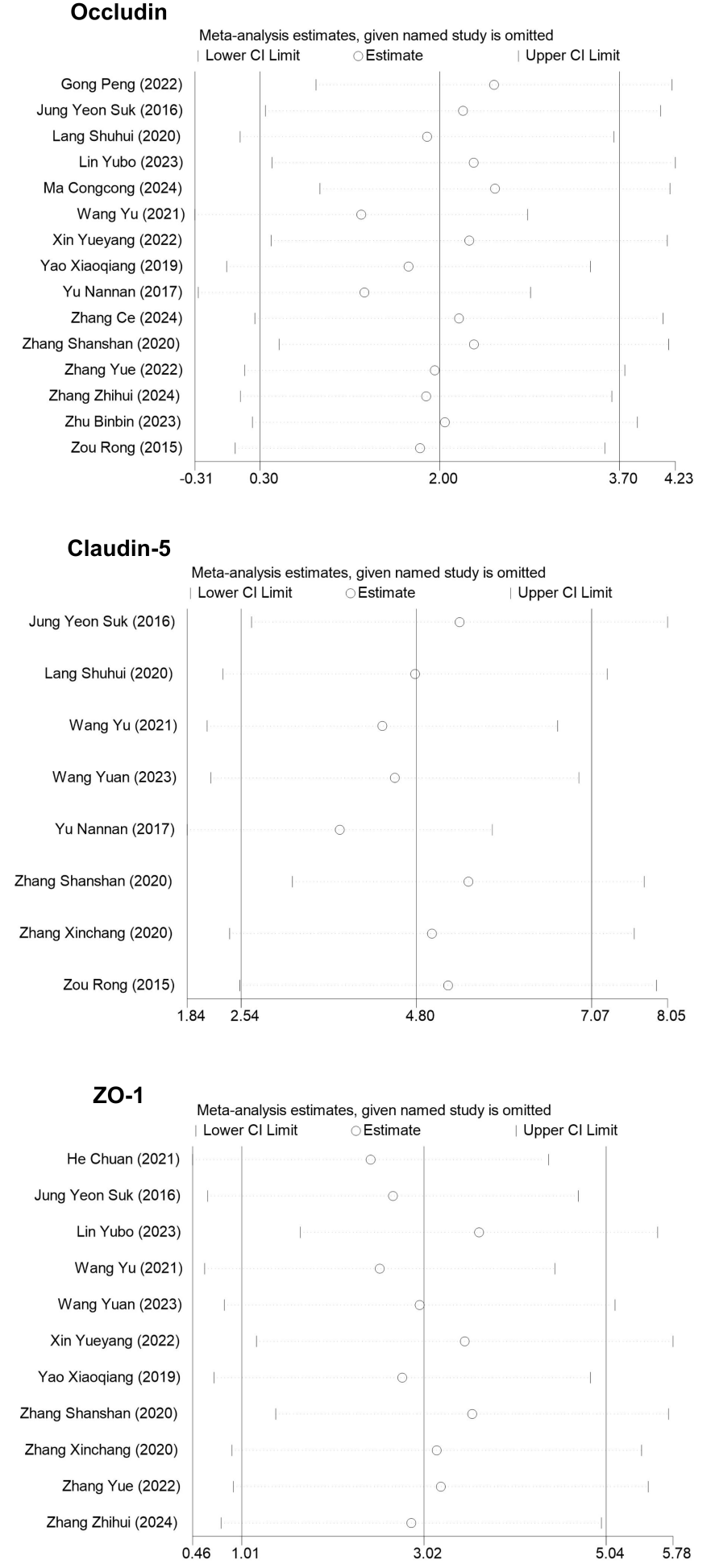


**Supplementary Figure 3.** Sensitivity analysis for the expression of tight junction proteins. (A) Occludin. (B) Claudin-5. (C) ZO-1.


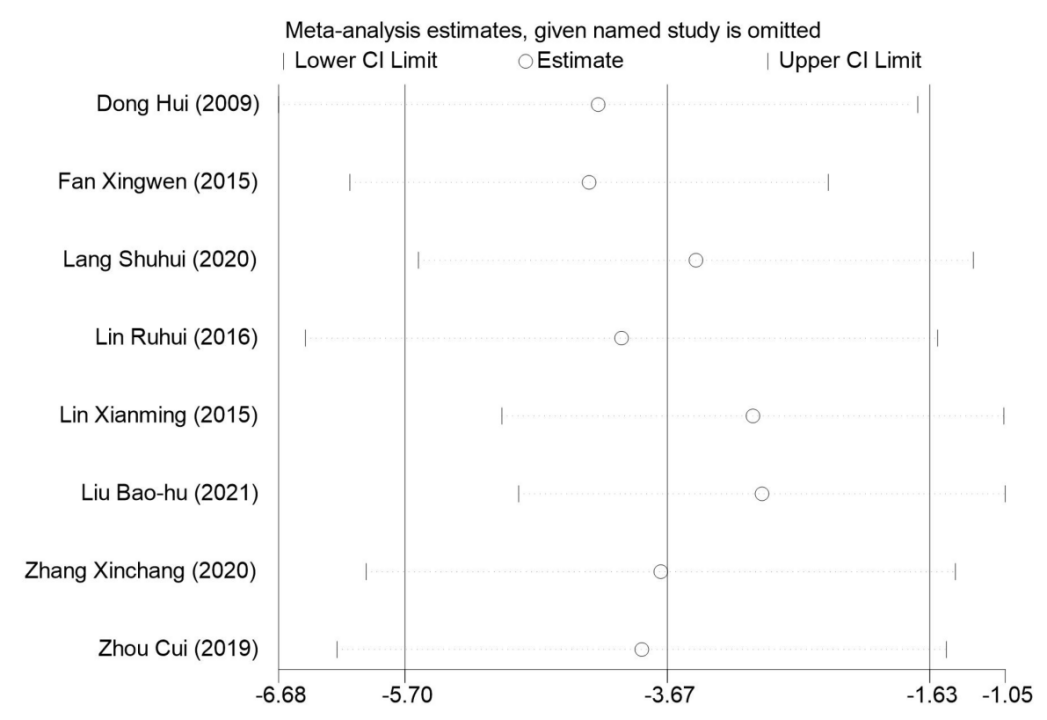


**Supplementary Figure 4.** Sensitivity analysis for the expression of Matrix Metalloproteinase-9 (MMP-9).


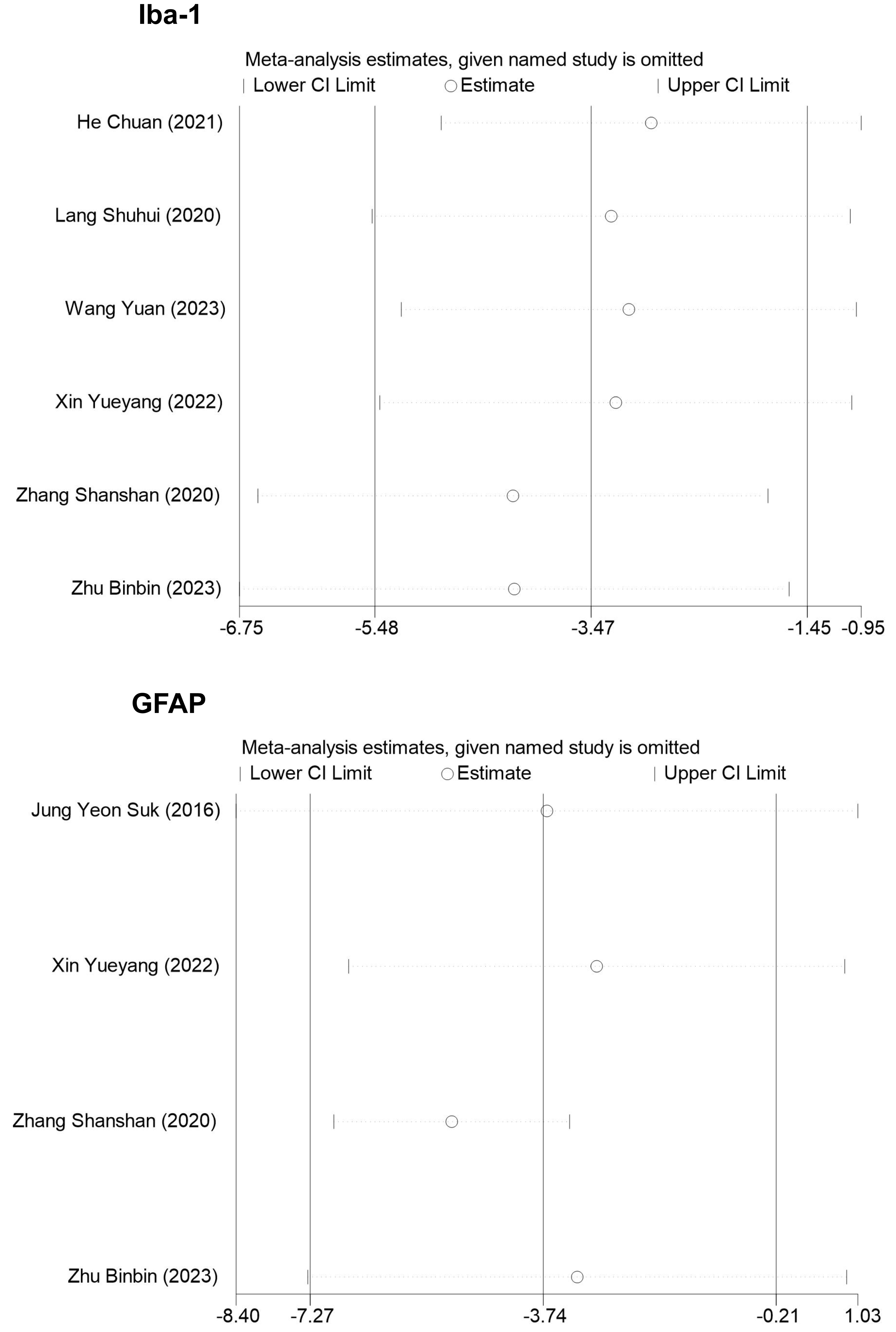


**Supplementary Figure 5.** Sensitivity analysis for the expression of glial activation markers. (A) Iba-1. (B) GFAP.


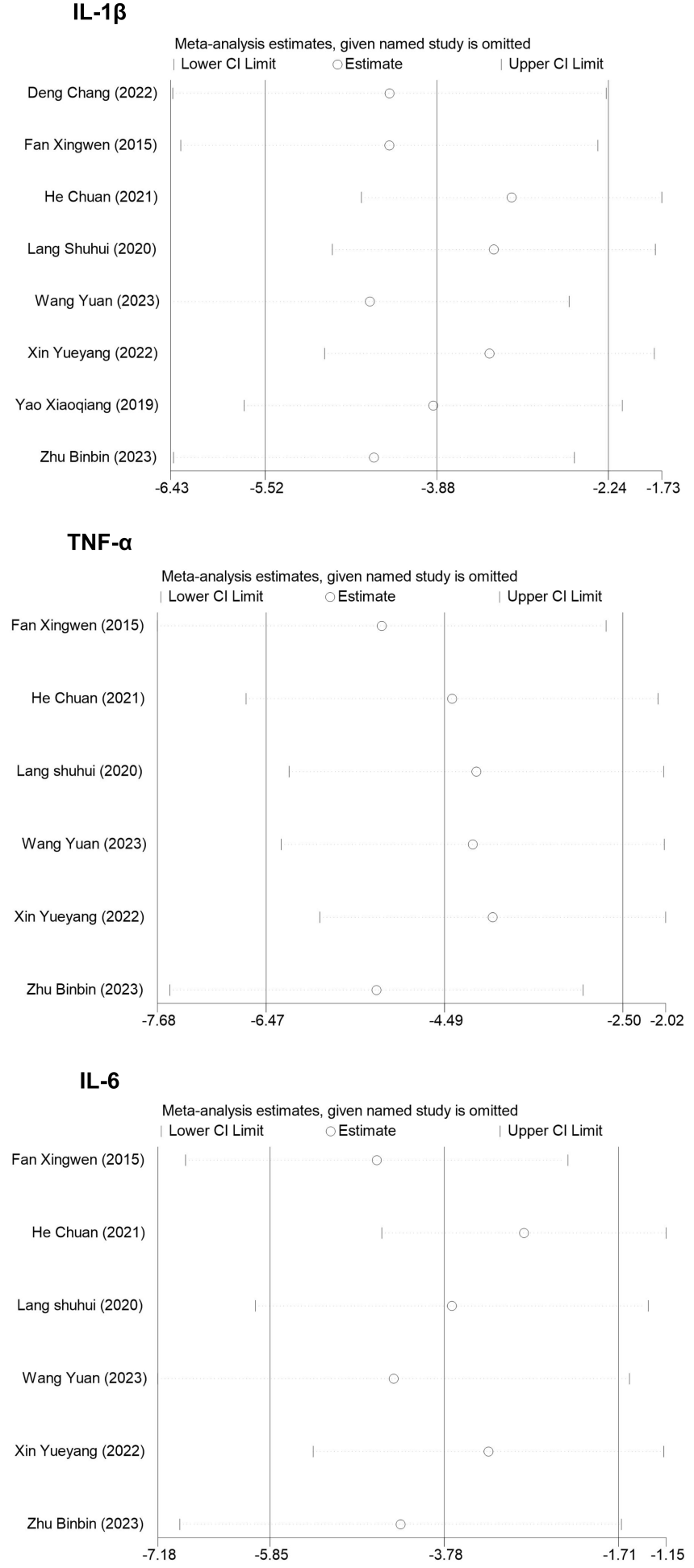


**Supplementary Figure 6.** Sensitivity analysis for the expression of inflammatory mediators. (A) IL-1β. (B) TNF-α. (C) IL-6.


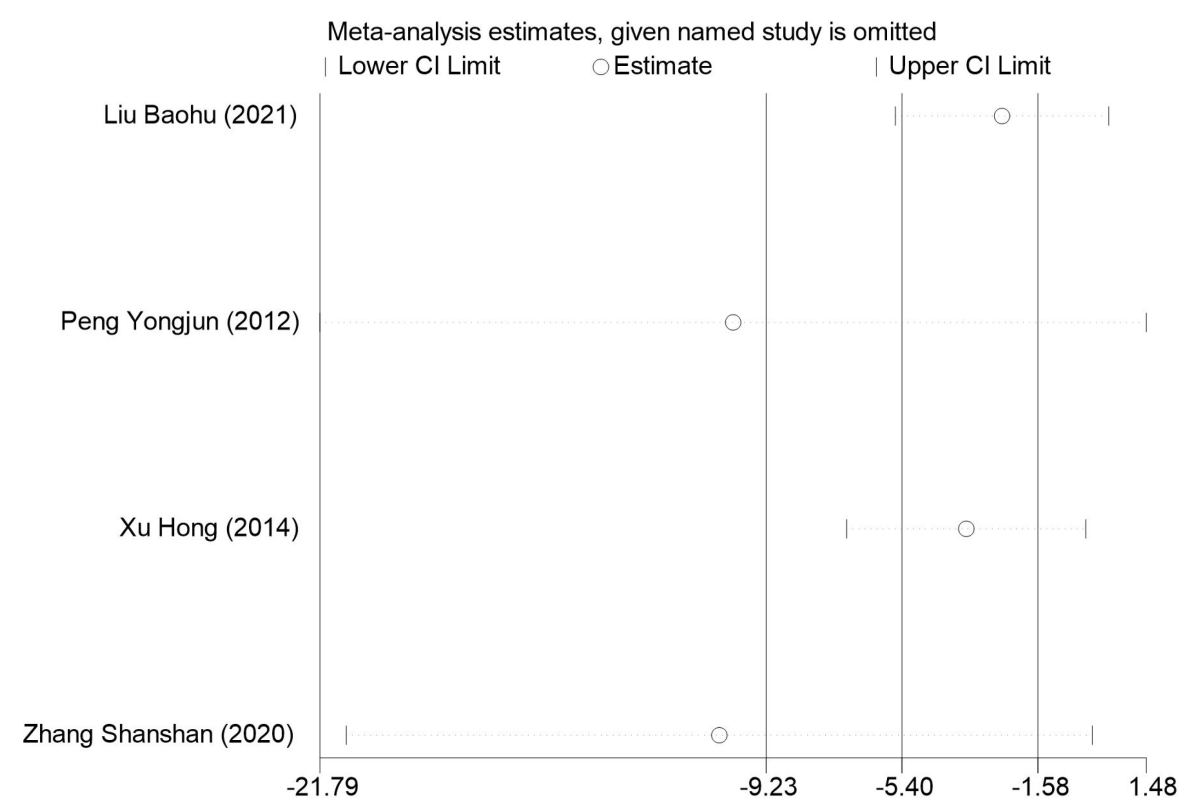


**Supplementary Figure 7.** Sensitivity analysis for the expression of Aquaporin-4 (AQP4).
