# Supplementary material for: Acupuncture improves blood–brain barrier integrity through multi-targeted mechanisms: a preclinical meta-analysis
Source: Front Neurol. 2025 Nov 7;16:1648117. doi: 10.3389/fneur.2025.1648117 (PMC12636094; doi:10.3389/fneur.2025.1648117)
Supplement: Supplementary file 5 [file Data_Sheet_2.pdf]

Supplementary Table 4 Detailed Characteristics

Study	Study Location	Animal	Weight (g)	Model	Acupoints	Acupuncture Protocol	Single Inter-Vention time	Inter-Vention times	Outcome	Acupuncture parameters
Deng Chang 2022	China	SD/♂	200 ±20	VD	GV20,GV14, BL23	EA 6/w, 4w	30min	24	EB; IL-1β	10/50HZ, SDW, 1-2mA
Dong Hui 2009	China	SD/♂	280 -300	MCAO	GV20	EA 1/d, 5d	30min	5	EB; MMP-9	15H，CW, 1 mA
Fan Xingwen 2015	China	SD/♂	NR	BI	GV20,GV26	EA 1/d, 1d	30min	1	EB; MMP-9 mRNA; IL-1β, TNF-α & IL-6 mRNA	2/15 Hz，SDW, 3 mA
Gong Peng 2022	China	SD/♂	NR	Aging rats	GV26, ST2	EA 1/d, 1d	40min	1	EB; Occludin	2/100 Hz, SDW, 3 mA
He Chuan 2021	China	SD/♂	360 ± 20	MCAO	GV20, ST36	EA 6/w, 8w	20min	48	ZO-1; Iba-1; IL-1β, TNF-α, IL-6	50HZ, CW, 1mA
Jung Yeon Suk 2016	South Korea	C57*BL/6J*/♂	20 - 25	MCAO	GV20, GV14	EA 1/d, 3d	20min	3	EB; occludin, claudin-5, ZO-1; GFAP	2Hz, CW, 1 mA
Lang shuhui 2020	China	SD/♂	300 - 380	SAH	GV20, GV14	EA 1/d, 3d	30min	3	EB; occludin, claudin-5; MMP-9; Iba-1;IL-1β, TNF-α, IL-6	2/15HZ, SDW, 2 mA
Li Huiqin 2016	China	SD/♂	NR	ICH	GV20, GB7	EA 1/d, 8d	30min	8	EB	2HZ, CW, 0.2mA
Lin Ruhui 2016	China	SD/♂	250 - 280	MCAO	GV20, GV24	EA 1/d, 7d	30min	7	MMP-9	1/20HZ, DSW, 1-3mA
Lin Xianming 2015	China	SD/♂	270 ± 20	MCAO	GV20, GV26	EA 1/d, 15d	30min	15	MMP-9	2/15HZ, SDW, 1 mA
Lin Yubo 2023	China	SD/♂	300 - 350	MCAO	GV20, GV26	EA 1/d, 1d	40min	1	EB; Occludin, ZO-1	2/100Hz, SDW, 3 mA
Liu Baohu 2021	China	SD/♂	280 - 320	TBI	Twelve Jing-Well Points	BL 2/d, 2d	NR	4	EB; MMP9; AQP4	The depth is 1 mm, and blood is extruded from each point (15–20 μL).
Lu Xuan 2013	China	Wistar	NR	MCAO	Twelve Jing-Well Points	BL 2/d, 3d	NR	6	EB	The depth is 1 mm, and the point is pressed until the bleeding stops.
Ma Congcong 2024	China	SD/♂	180 - 220	PT	GV20, GV26	EA 1/d, 1d	40min	1	EB; Occludin	2/100Hz, SDW, 3 mA
Peng Yongjun 2012	China	SD/♂	215 - 230	MCAO	GV20, GV26	EA 1/d, 1d	30min	1	AQP4	3.85/6.25HZ, SDW, 0.8 – 1.0 mA
Wang Yu 2021	China	SAMP8/ ♀/♂ mix	25 ± 2	AD	CV17, CV12, CV6, ST36, SP10	MA 6/w, 4w	30s	24	EB; Occludin, claudin-5, ZO-1	SP10: Depth 2-3 mm, frequency <60 min^{-1}; CV17, CV12, CV6, ST36: Depth 2-3 mm, frequency >120 min^{-1}.

Study	Study Location	Animal	Weight (g)	Model	Acupoints	Acupuncture Protocol	Single Inter-Vention time	Inter-Vention times	Outcome	Acupuncture parameters
Wang Yuan 2023	China	SAMP8/♂	NR	AD	GV29, LI20	EA 5/w, 4w	20min	20	claudin-5, ZO-1; Iba-1; IL-1β, TNF-α, IL-6	2/15HZ, SDW, 1 mA
Wu Xudong 2001	China	SD/♂	260 ± 20	MCAO	GV20, GV26	EA 1/d, 1d	60min	1	EB	20/4HZ, SDW, 3 mA
Xin Yueyang 2022	China	SD/♂	200 - 220	SAE	GV20, ST36	EA 1/d, 4d	20min	4	Occludin, ZO-1; Iba-1, GFAP; IL-6, IL-1β, TNF-α	15HZ, CW, 1 mA
Xu Hong 2014	China	SD/♂	230 - 250	CIRI	GV20, ST36	EA 1/d, 2d	20min	2	AQP4	2Hz, CW, 1 mA
Yao Xiaoqiang 2019	China	SD/♂	200 ± 20	MCAO	Anterior Temporal Line	MA 1/d, 6d	30min	6	EB; Occludin & ZO-1 mRNA; IL-1β	100 rpm for 1 minute, once every 10 minutes.
Yu Bin 2011	China	KM/♀/♂ mix	18 - 22	Nomal	GV20, GV15	EA 1/d, 1d	20min	14	EB	2Hz, CW, 1 mA
Yu Nannan 2017	China	Wistar,♂	220 - 250	pMCAO	Twelve Jing-Well Points	BL 1/d, 4d	NR	4	EB; Occludin & claudin-5 mRNA	The depth is 1 mm, and blood is extruded from each point (15–20 μL).
Zhang Ce 2024	China	SD/♂	250 ± 20	ICH	GV20, GB7	MA 1/d, 7d	30min	7	EB; Occludin	Three twisting maneuvers, each lasting 5 minutes at 200 ± 10 rpm.
Zhang Jiangsong 2018	China	SD/♂	250 - 280	MCAO	GV20, GV26	EA 1/d, 1d	8min	1	EB	100HZ, CW, 2 mA
Zhang Shanshan 2020	China	SD/♂	NR	Nomal	GV20, GV26	EA 1/d, 1d	40min	1	EB; Occludin, claudin-5, ZO-1; Iba1, GFAP; AQP4	2/100 Hz, SDW, 3 mA
Zhang Xinchang 2020	China	SD/♂	320 ± 20	MCAO	GV26, PC6	EA 1/d, 1d	30min	1	EB; claudin-5, ZO-1; MMP-9	2/15HZ, SDW, 1 mA
Zhang Yue 2022	China	*APP/PS1/* ♂	30 ± 2	AD	GV20, GV29, ST36	MA 1/d, 38d	20min	38	EB; Occludin, ZO-1	Bidirectional twisting at 180°/s within 90°, performed every 5 minutes for 15 seconds.
Zhang Zhihui 2024	China	SD/♂	320 ± 20	MCAO	GV20, PC6	MA 1/d, 1d	30 min	1	EB; Occludin, ZO-1	GV26: Sparrow-pecking manipulation technique PC6:120-160r/min
Zhou Cui 2019	China	SD/♂	180 - 220	AD	GV20, BL23, GV29	Mox 1/d, 21d	10 min	21	EB; MMP-9	Mild moxibustion was applied 2-3 cm above the acupoint.
Zhu Binbin 2023	China	*APP/PS1/* ♂	30 - 40	POCD	GV20, GV14, ST36, LI11	EA 1/d, 9d	30min	9	Occludin;Iba-1, GFAP; IL-1β, TNF-α, IL-6	15HZ, CW,

Study	Study Location	Animal	Weight (g)	Model	Acupoints	Acupuncture Protocol	Single Inter-Vention time	Inter-Vention times	Outcome	Acupuncture parameters
Zou Rong 2015	China	SD/♂	280 - 320	MCAO	GV20	EA 1/d, 5d	30min	5	Occludin, claudin- 5	2/15HZ, SDW, 1 mA

Note: VD: Vascular Dementia, MCAO: Middle Cerebral Artery Occlusion Model, BI: Brain X-ray Radiation, SAH: Subarachnoid Hemorrhage, ICH: Spontaneous Intracerebral Hemorrhage, TBI: Traumatic Brain Injury, PT: C6 Glioma Model, AD: Alzheimer's Disease, SAE: Sepsis-Associated Encephalopathy, CIRI: Cerebral Ischemia/Reperfusion Injury, pMCAO: Permanent Middle Cerebral Artery Occlusion, POCD: Postoperative Cognitive Dysfunction, CW: Continuous Wave, SDW: sparse-dense wave, EB: Evans Blue, EA:Electroacupuncture, MA: Manual acupuncture, Mox: moxibustion, KM:Kun ming mice, BL: Bloodletting, NR: No mention was made of this in the study.
